# Burden of chronic respiratory disease in Fujian province, 1990–2021

**DOI:** 10.1371/journal.pone.0335352

**Published:** 2025-11-14

**Authors:** Yanrong Yin, Xiuquan Lin, Wenling Zhong, Tiehui Chen, Jingyu Chen

**Affiliations:** Department of Chronic and Noncommunicable Disease Control and Prevention, Fujian Provincial Center for Disease Control and Prevention, Fuzhou, Fujian province, People’s Republic of China; University of Zagreb School of Medicine: Sveuciliste u Zagrebu Medicinski fakultet, CROATIA

## Abstract

**Background:**

Chronic respiratory diseases (CRDs) are major public health problems with significant risk factors. This study aimed to analyze the disease burden and attributable risk factors of CRDs in Fujian, Southeast China, between 1990 and 2021 and provide a scientific basis for developing public health policies for Fujian government.

**Methods:**

Data on incidence, prevalence, deaths, disability-adjusted life years (DALYs), and attributable risk factors for CRDs among Fujian residents were estimated as part of the Global Burden of Disease 2021 (GBD 2021) study at the provincial level. Joinpoint regression was used to assess the long-term temporal trends of the disease burden and identify the main risk factors of CRD between 1990 and 2021.

**Results:**

In 1990, there were 0.22 million (95% *CI:* 0.20–0.28) new CRD cases and 1.56 million (95% *CI:* 1.38–1.78) patients with CRDs, increasing up to 0.24 million (95% *CI:* 0.21–0.28) new cases and 2.00 million (95% *CI:* 1.80–2.24) patients with CRDs in 2021. However, the age-standardized incidence rate, age-standardized prevalence rate, age-standardized mortality rate, and age-standardized DALY rate decreased by 29.63%, 29.26%, 75.92%, and 73.62% in Fujian (all *P* < 0.01) in these 32 years. The DALYs for CRDs were higher in males, patients older than 50 years, and patients with COPD. Smoking, ambient particulate matter pollution, and occupational exposure were the top three attributable risk factors of DALYs for CRDs in Fujian in 2021. Moreover, the proportion attributed to household air pollution from solid fuels significantly decreased.

**Conclusion:**

The total CRD burden in Fujian province decreased, but the number of new cases and patients continuously increased. The ranking of risk factors of CRDs has changed between 1990 and 2021. Older men, smokers, and people with occupational exposure were the key groups that relieved the CRD burden. Efforts should focus on quitting smoking and reducing air pollution.

## Introduction

Chronic respiratory diseases (CRDs) are a group of long-term diseases characterized by structural or functional abnormalities of the lungs and airways [1]. Common CRDs include chronic obstructive pulmonary disease (COPD), asthma, interstitial lung disease and pulmonary sarcoidosis (ILDPS), pneumoconiosis, and other CRDs [1,2]. CRDs are incurable, and patients often experience respiratory symptoms, such as dyspnea, cough, sputum production, and acute exacerbation [1]. In 2021, the global prevalence of CRDs was estimated at 468 million cases, with approximately 4.41 million deaths, making CRDs the fourth leading cause of death worldwide [2,3]. In China, CRDs ranked as the third leading cause of death, with an estimated 1.33 million deaths annually [2,3]. The main risk factors for CRDs were smoking, air pollution, and occupational exposure [1,2]. As one of the four major chronic diseases globally, CRDs impose a significant burden on public health due to their high mortality and disability rates, which severely compromise the quality of life and place a substantial economic and social strain on patients and their families [4].

The “Healthy China Initiative (2019–2030)” and the “Medium and Long-Term Plan for Chronic Diseases in Fujian Province (2017–2025)” have clearly outlined specific targets, including “reducing the premature mortality rate due to major chronic diseases and effectively controlling the disease burden of chronic diseases” [2,5,6]. Recently, the “Healthy China Initiative-Implementation Plan for the Prevention and Control of CRDs (2024–2030)” has also outlined specific goals for public health strategies aimed at preventing and controlling CRDs [2,7].

To date, reports on the burden of CRDs in Fujian province are lacking. This study used GBD 2021 data to analyze the changes in the burden and attributable risk factors of CRDs among Fujian residents between 1990 and 2021.

## Materials and methods

### Source of materials

The GBD 2021 database can be downloaded and obtained through the Global Health Data Exchange website (https://vizhub.healthdata.org/gbd-results) [2]. It provides the most comprehensive disease burden indicators, including incidence, prevalence, mortality, disability-adjusted life years (DALYs), and attributable risk factors. The database encompasses 371 diseases or injuries in 204 countries and regions, spanning from 1990 to 2021, and employs a unified and comparable methodology. It also estimates the attributable disease burden of 88 risk factors, stratified by age, sex, and year [2]. The data of GBD 2021 of Fujian province was as a part of China GBD 2021. GBD information is anonymous and publicly available for download, does not collect personal data, and does not require ethical approval for use.

### Analytical indicators

The incidence rate, prevalence rate, mortality rate, and DALYs for CRDs worldwide, in China, and in Fujian province were used as health indicators for measuring the burden of CRDs. DALYs refer to all the healthy life years lost from the onset of the disease to death. It includes two parts: years of life lost (YLLs) due to premature death and years of life lost due to disability (YLDs). The DALY estimates from the GBD 2021 analysis, which were used for this study, incorporate the standard GBD comorbidity adjustment to prevent the double-counting of disability.

GBD 2021 adopted the concept of population-attribution fraction (PAF) of DALYs, which is defined as quantifying the magnitude of the pathogenic or lethal effect of a certain risk factor on the population. We utilized the proportions of eight attributable risk factors to that contributed to DALYs and deaths from CRDs, namely smoking, occupational exposure, ambient particulate matter (PM) pollution, household air pollution from solid fuels, secondhand smoke, ambient ozone pollution, low temperature, and high temperature,which were processed by GBD 2021 team [8].

### Statistical analysis

We conducted a descriptive data analysis of the disease burden and various indicators among residents of Fujian province between 1990 and 2021 using Excel 2017 software. Age-standardized rates (ASRs; rates per 100,000) represented the disease burden, including the age-standardized incidence rate (ASIR), age-standardized prevalence rate (ASPR), age-standardized mortality rate (ASMR), age-standardized DALYs rate (ASDR), age-standardized YLLs rate (ASYLLR), and age-standardized YLDs rate (ASYLDR). The ASRs were calculated based on the global standard population age structure in the GBD 2021 report.

The Joinpoint 5.1.0 software developed by the National Cancer Institute of the United States was used, and a log-linear model was employed to calculate the annual percent change (APC) and the average annual percent change (AAPC). An annual trend analysis was conducted on the AAPC and its 95% confidence interval (95% *CI*). The changing ASIR, ASPR, ASMR, and ASDR trends of CRDs in the Fujian province were statistically analyzed. A two-sided significance level (α) of 0.05 was adopted.

## Results

### Changes in the CRD disease burden in Fujian between 1990 and 2021

In 2021, 241,441 (95% *CI:* 210,978–284,998) new cases of CRDs were reported among Fujian residents, which was 5.28% higher than in 1990. The total number of patients with CRDs in 2021 in Fujian was 2.00 million (95% *CI:* 1.80–2.24), an increase of 28.06% compared to that in 1990. There were 22,532 (95% *CI:* 17,232–28,787) CRD-related deaths in 2021 and 27,564 (95% *CI:* 22,192–32,928) deaths in 1990, indicating a decrease of 18.26% over the past 32 years.

The DALYs for CRDs in Fujian was 0.47-million-person years (95% *CI:* 0.38–0.57), decreasing by 26.03% compared with that in 1990. The YLLs for CRDs in Fujian in 2021 were 0.34 million (95% *CI:* 0.26–0.44) person-years, with a decrease of 37.61%. The YLDs during the same period were 0.13 million (95% *CI:* 0.10–0.16) person-years, increasing by 46.87% compared to that in 1990.

In 2021, the ASIR for CRDs among Fujian residents was 601.15 per 100,000 (95% *CI:* 511.05–737.42), decreasing by 29.63% compared to that in 1990. The AAPC of ASIR between 1990 and 2021 was −1.14% (95% *CI:* −1.25 to −1.04), and it was statistically significant. The ASPR for CRDs in 2021 was 4,391.81 per 100,000 (95% *CI:* 3875.90–5014.08), decreasing by 29.26% compared to that in 1990. The AAPC of ASPR between 1990 and 2021 was −1.12% (95% *CI:* −1.19 to −1.05). The ASMR for CRDs in Fujian in 2021 was 50.53 per 100,000 (95% *CI:* 38.36–64.36), decreasing by 75.93% over 32 years. The AAPC of ASMR was −4.51% (95% *CI:* −5.04 to −3.98) during the same period.

The ASDR for CRDs in Fujian in 2021 was 980.97 per 100,000 (95% *CI:* 804.50–1195.49), decreasing by 73.62%. The AAPC of ASDR was −4.23% (95% *CI:* −4.52 to –3.93). Precisely, the ASYLLR reduced from 3,350.00 per 100,000 in 1990 to 712.56 per 100,000 in 2021, a decrease of 78.73%, with an AAPC of −4.89 (95% *CI:* −5.33 to −4.45). The ASYLDR decreased from 368.76 per 100,000 in 1990 to 268.41 per 100,000 in 2021, a decrease of 27.21%, with an AAPC of −1.02 (95% *CI:* −1.05 to −0.99, Table 1).

**Table 1:** The change of disease burden of CRD by sex in Fujian, 1990-2021.

Index		Numbers (95%CI)				Number Change(%)	ASR (/10^6) (95%CI)				ASR change(%)	AAPC% (95%CI)
		1990		2021			1990		2021			
Total	Incidence	229326.16	(195212.65, 276912.52)	241441.24	(210977.54, 284997.66)	5.28	854.25	(751.18, 1000.72)	601.15	(511.05, 737.42)	-29.63	-1.14(-1.25, -1.04)
	Prevalence	1558737.40	(1384106.36, 1784261.70)	1996050.04	(1800228.22, 2240507.71)	28.06	6208.44	(5651.06, 6909.10)	4391.81	(3875.90, 5014.08)	-29.26	-1.12(-1.19,-1.05)
	Mortality	27564.18	(22191.78, 32927.91)	22532.28	(17232.28, 28786.90)	-18.26	209.87	(170.47, 248.84)	50.53	(38.36, 64.36)	-75.92	-4.51(-5.04,-3.98)
	DALYs	634371.67	(524863.85, 758595.76)	469265.27	(382978.21, 569740.39)	-26.03	3718.76	(3079.45, 4389.72)	980.97	(804.50, 1195.49)	-73.62	-4.23(-4.52,-3.93)
	YLLs	547382.17	(442014.10, 659947.25)	341502.59	(260884.15, 439582.02)	-37.61	3350.00	(2707.64, 4000. 28)	712.56	(544.04, 914.98)	-78.73	-4.89(-5.33, -4.45)
	YLDs	86989.50	(66812.41, 110519.75)	127762.69	(100064.06, 157143.12)	46.87	368.76	(289.54, 456.23)	268.41	(209.66, 333.90)	-27.21	-1.02(-1.05,-0.99)
Males	Incidence	126355.20	(106282.46, 156446.02)	135589.49	(117804.82, 162297.49)	7.31	932.07	(817.28, 1108.55)	668.60	(569.52, 822.82)	-28.27	-1.07(-1.18, -0.97)
	Prevalence	817859.76	(711781.77, 951883.71)	1034316.19	(926578.50, 1164403.64)	26.47	6428.56	(5779.39, 7262.55)	4597.77	(4053.32, 5305.28)	-28.48	-1.08(-1.16, -1.00)
	Mortality	15030.50	(11600.88, 19287.00)	14511.45	(10709.30, 19404.90)	-3.45	297.47	(244.31, 361.14)	82.09	(59.78, 107.92)	-72.40	-4.15(-4.53, -3.77)
	DALYs	363178.28	(284801.37, 458810.08)	286500.46	(218225.96, 368207.21)	-21.11	4966.79	(4005.65, 6130.03)	1360.18	(1051.91, 1717.32)	-72.61	-4.19(-4.58, -3.79)
	YLLs	322716.67	(247038.13, 419353.74)	230145.73	(166825.12, 314103.15)	-28.68	4634.18	(3688.98, 5825.50)	1117.26	(828.10, 1495.19)	-75.89	-4.58(-5.01, -4.15)
	YLDs	40461.61	(30003.76, 53549.94)	56354.74	(43565.98, 69638.56)	39.28	332.62	(253.98, 427.21)	242.92	(185.70, 304.23)	-26.97	-1.01(-1.07, 0.96)
Females	Incidence	102970.97	(88541.12, 123173.29)	105851.74	(92960.15, 124318.19)	2.80	781.22	(689.85, 910.72)	531.55	(451.36, 649.37)	-31.96	-1.26(-1.43, -1.10)
	Prevalence	740877.64	(661524.32, 839150.95)	961733.85	(861491.72, 1075140.92)	29.81	5959.20	(5421.63, 6607.51)	4138.45	(3670.10, 4708.37)	-30.55	-1.85(-1.27, -1.10)
	Mortality	12533.68	(8984.40, 16053.30)	8020.83	(5447.42, 11650.24)	-36.01	164.05	(118.83, 209.04)	30.74	(20.85, 44.61)	-81.26	-5.28(-5.97, -4.59)
	DALYs	271193.39	(205983.97, 339839.39)	182764.81	(142944.64, 238855.29)	-32.61	2905.91	(2190.52, 3637.43)	708.56	(557.55, 925.15)	-75.62	-4.46(-5.08, -3.84)
	YLLs	224665.50	(161159.37, 291699.32)	111356.86	(76323.15, 163505.22)	-50.43	2511.30	(1808.34, 3230.45)	420.59	(288.11, 618.50)	-83.25	-5.62(-6.19, -5.05)
	YLDs	46527.89	(35327.27, 58537.99)	71407.95	(54888.16, 88306.42)	53.47	394.61	(306.20, 482.56)	287.97	(220.62, 362.78)	-27.02	-1.01(-1.09, -0.94)

Note: CRD: chronic respiratory disease; DALY: disability-adjusted life years; YLD: years of life lost; YLL: years lived with disability;

ASR: age standardized rate; AAPC: average annual percentage change; CI: confidence interval.

### Changes in CRD burden in different sexes

From 1990 to 2021, the number of new cases and the total number of patients with CRDs among males and females in Fujian showed an upward trend. Approximately 1.03 million (95% *CI:* 0.93–1.16) male patients had CRDs in 2021, representing a 26.47% increase from the number in 1990. More males had CRDs than females, with 0.96 million (95% *CI:* 0.86–1.08) patients in 2021, representing a 29.81% increase from the 1990 prevalence. Meanwhile, the number of deaths, DALYs, and YLLs among both males and females decreased. The number of YLDs by women increased by 53.47% between 1990 and 2021, which was higher than the increase among men (39.28%).

Compared to the 1990 data, the ASIR, ASPR, ASMR, and ASDR in 2021 exhibited decreasing trends in both sexes. The ASIR was 668.60/100,000 in males and 531.55/100,000 in females in 2021. Compared with 1990, the ASIR decreased by 28.27% in males and 31.96% in females. The ASPR among men was 4597.77/100,000 and 4138.45/100,000 among women. Over the past 32 years, the ASPR of men decreased by 28.48%, and that of women decreased by 30.55%. In 2021, the ASMR was 82.09/100,000 in males and 30.74/100,000 in females. The ASMR decreased by 72.40% in males and 81.26% in females. The ASDR was 1360.18/100,000 in males and 708.56/100,000 in females in 2021. Compared with 1990, the ASDR decreased by 72.61% in males and 75.62% in females. Precisely, the age-standardized YLLs and YLDs rates showed decreasing trends (Table 1).

### Changes in CRD burden in different age groups

The entire population was divided into five age groups. The numbers of new cases, patients, and deaths from CRDs among 0–5-year-olds, 5–14-year-olds, and 15–49-year-olds all showed decreasing trends. However, the number of new cases and patients with CRDs among 50–69-year-olds and those above 70 years old increased. Particularly among those above 70 years old, the incidence of CRDs increased from 26,055 cases (95% *CI:* 22,752–29,172) in 1990–63,674 cases (95% *CI:* 54,757–72,269) in 2021, and the prevalence of CRDs increased from 248,065 cases (95% *CI:* 220,217–274,074) in 1990–639,718 cases (95% *CI:* 549,005–725,022) in 2021, increasing by more than 2.5 times. The number of deaths from CRDs only increased in the above 70-year-old age group, with an increase of 0.68%.

The DALYs of all five age groups showed downward trends. The decrease in the under-5 age group was 62.76%, and this decline was the most significant decline among the age groups. The YLLs in the under-5 age group decreased by 95.26%, the greatest decline in YLLs. By 2021, the YLDs in the 50–69-year-old age group had increased by 69.11%, and those in the above 70-year-old age group had increased by 160.75%. After age standardization, the ASR indicators of CRDs in each age group of Fujian residents all showed decreasing trends between 1990 and 2021. ASIR, ASPR, and ASDR showed the most significant decrease in the 50–69 years age group, decreasing by 48.66%, 42.98%, and 81.05%, respectively. The ASMR in children under 5 years old declined more significantly, with a reduction of 93.75% (Table 2).

**Table 2:** The change of disease burden of CRD by age groups in Fujian, 1990-2021.

Disease	Index	Numbers (95%*CI)*				Number Change(%)	Rate (95%CI)				Rate change
		1990		2021			rate	(/10^6)	rate	(/10^6)	
0-5 years	Incidence	67955.98	(41806.61, 107307.45)	42545.79	(25147.67, 70613.23)	-37.39	2045.44	(1258.36, 3229.91)	1623.92	(959.86, 2695.21)	-20.61
	Prevalence	147155.23	(91752.27, 209874.64)	95619.34	(55457.90, 143599.57)	-35.02	4429.31	(2761.70, 6317.13)	3649.33	(2116.76, 5481.02)	-17.61
	Mortality	58.47	(39.06, 83.75)	2.77	(1.70, 4.58)	-95.26	1.76	(1.18, 2.52)	0.11	(0.06, 0.17)	-93.75
	DALYs	11184.96	(7711.92, 15610.45)	4164.86	(2173.63, 6975.32)	-62.76	336.66	(232.13, 469.87)	158.97	(82.96, 266.24)	-52.78
	YLLs	5181.04	(3457.17, 7420.95)	245.61	(150.84, 407.18)	-95.26	155.95	(104.06, 223.37)	9.37	(5.76, 15.54)	-93.99
	YLDs	6003.92	(3189.95, 9960.83)	3919.26	(1937.42, 6761.52)	-34.72	180.72	(96.02, 299.82)	149.59	(73.95, 258.08)	-17.23
5-14 years	Incidence	42807.47	(23162.52, 70161.96)	36402.13	(19470.25, 59245.64)	-14.96	680.42	(368.16, 1115.21)	632.08	(338.08, 1028.73)	-7.10
	Prevalence	299317.83	(202292.23, 453178.86)	231065.42	(153739.40, 354107.39)	-22.80	4757.59	(3215.39, 7203.18)	4012.18	(2669.51, 6148.67)	-15.67
	Mortality	26.85	(20.54, 34.48)	3.17	(2.40, 4.25)	-88.19	0.43	(0.33, 0.55)	0.06	(0.04, 0.07)	-86.05
	DALYs	14319.01	(8826.14, 23794.04)	9683.10	(5267.62, 17622.37)	-32.38	227.60	(140.29, 378.20)	168.14	(91.47, 305.99)	-26.13
	YLLs	2154.25	(1646.67, 2771.43)	254.96	(191.66, 340.95)	-88.17	34.24	(26.17, 44.05)	4.43	(3.33, 5.92)	-87.07
	YLDs	12164.76	(6696.96, 21711.68)	9428.15	(5037.55, 17357.14)	-22.50	193.36	(106.45, 345.10)	163.71	(87.47, 301.39)	-15.33
15-49years	Incidence	49519.48	(38875.17, 62545.55)	37754.24	(29956.04,44751.45)	-23.76	297.97	(233.92, 376.35)	183.42	(145.54, 217.42)	-38.44
	Prevalence	476282.45	(395703.30, 563805.17)	417629.20	(347119.81, 490867.80)	-12.31	2865.92	(2381.05, 3392.57)	2028.98	(1686.42, 2384.80)	-29.20
	Mortality	806.19	(624.12, 1017.55)	294.50	(217.43, 398.90)	-63.47	4.85	(3.76, 6.12)	1.43	(1.06, 1.94)	-70.52
	DALYs	66727.46	(55038.23, 80547.42)	39411.18	(31781.29, 47942.28)	-40.94	401.52	(331.18, 484.68)	191.47	(154.40, 232.92)	-52.31
	YLLs	41221.50	(32169.11, 51901.21)	14335.97	(10715.14, 19262.49)	-65.22	248.04	(193.57, 312.30)	69.65	(52.06, 93.58)	-71.92
	YLDs	25505.95	(18435.42, 33971.65)	25075.21	(18763.05, 32499.37)	-1.69	153.48	(110.93, 204.42)	121.82	(91.16, 157.89)	-20.63
50-69years	Incidence	42987.76	(36698.79, 49505.58)	61064.93	(51655.06, 71321.68)	42.05	1162.97	(992.83, 1339.30)	597.04	(505.04, 697.32)	-48.66
	Prevalence	387917.03	(346038.68, 432664.10)	612027.26	(529829.02, 694888.88)	57.77	10494.54	(9361.58, 11705.11)	5983.89	(5180.22, 6794.04)	-42.98
	Mortality	7446.65	(5772.51, 9427.29)	2874.32	(2072.00, 4031.96)	-61.40	201.46	(156.17, 255.04)	28.10	(20.26, 39.42)	-86.05
	DALYs	236786.26	(188027.61, 292715.79)	124147.67	(97926.52, 158109.40)	-47.57	6405.91	(5086.82, 7919.01)	1213.81	(957.44, 1545.86)	-81.05
	YLLs	211030.44	(163563.43, 266671.67)	80592.01	(57926.30, 112204.47)	-61.81	5709.13	(4424.97, 7214.42)	787.96	(566.35, 1097.04)	-86.20
	YLDs	25755.82	(20506.99, 31531.76)	43555.67	(33865.57, 53168.04)	69.11	696.79	(554.79, 853.05)	425.85	(331.11, 519.83)	-38.88
>70 years	Incidence	26055.46	(22751.55, 29171.90)	63674.14	(54757.12, 72269.39)	144.38	2919.20	(2549.03, 3268.36)	2232.28	(1919.67, 2533.61)	-23.53
	Prevalence	248064.87	(220216.50, 274074.21)	639717.82	(549005.39, 725022.32)	157.88	27792.65	(24672.58, 30706.68)	22427.12	(19246.94, 25417.71)	-19.31
	Mortality	19226.03	(15625.37, 22728.96)	19357.52	(14689.00, 24600.37)	0.68	2154.04	(1750.63, 2546.50)	678.63	(514.96, 862.44)	-68.50
	DALYs	305353.98	(249726.28, 358173.50)	291858.45	(232810.21, 358908.56)	-4.42	34211.20	(27978.79, 40128.98)	10231.92	(8161.82, 12582.55)	-70.09
	YLLs	287794.94	(231816.92, 342026.49)	246074.05	(187536.30, 314091.28)	-14.50	32243.92	(25972.26, 38319.90)	8626.82	(6574.62, 11011.36)	-73.25
	YLDs	17559.04	(13980.14, 21197.50)	45784.40	(34845.32, 56140.14)	160.75	1967.28	(1566.30, 2374.92)	1605.10	(1221.60, 1968.15)	-18.41

Notes: CRD: chronic respiratory disease; DALY: disability-adjusted life years; YLD: years of life lost; YLL: years lived with disability;

CI: confidence interval.

### Changes in the CRD burden attributable to five diseases

According to GBD 2021, CRDs are classified into five major categories, including COPD, asthma, pneumoconiosis, ILDPS, and other CRDs. From 1990 to 2021, the incidence and prevalence of CRDs increased. The incidence and prevalence of COPD, pneumoconiosis, and ILDPS increased by 88.77% and 105.72%, 48.82% and 106.97%, and 197.35% and 182.33%, respectively. In contrast, the incidence and prevalence of asthma decreased by 20.13% and 13.80%, respectively. The ASIR, ASPR, ASMR, and ASDR for CRDs, COPD, pneumoconiosis, and asthma all showed statistically significant decreasing trends. During these 32 years, the ASIR and ASPR of ILDPS increased by 18.80% and 8.67%, respectively (Table 3).

**Table 3:** The change of disease burden of CRD by categories in Fujian, 1990-2021.

Disease	Index	Numbers (95%*CI*)				Number Change(%)	ASR(/10^6) (95%*CI)*				ASR Change	AAPC% (95%CI)
		1990		2021			1990		2021			
All CRD	Incidence	229326.16	(195212.65, 276912.52)	241441.24	(210977.54, 284997.66)	5.28	854.25	(751.18, 1000.72)	601.15	(511.05, 737.42)	-29.63	-1.14 (-1.25,-1.04)
	Prevalence	1558737.40	(1384106.36, 1784261.70)	1996050.04	(1800228.22, 2240507.71)	28.06	6208.44	(5651.06, 6909.10)	4391.81	(3875.90, 5014.08)	-29.26	-1.12(-1.19,-1.05)
	Mortality	27564.18	(22191.78, 32927.91)	22532.28	(17232.28, 28786.90)	-18.26	209.87	(170.47, 248.84)	50.53	(38.36, 64.36)	-75.92	-4.51 (-5.04,-3.98)
	DALYs	634371.67	(524863.85, 758595.76)	469265.27	(382978.21, 569740.39)	-26.03	3718.76	(3079.45, 4389.72)	980.97	(804.50, 1195.49)	-73.62	-4.23(-4.52,-3.93)
	YLLs	547382.17	(442014.10, 659947.25)	341502.59	(260884.15, 439582.02)	-37.61	3350.00	(2707.64, 4000. 28)	712.56	(544.04, 914.98)	-78.73	-4.89(-5.33, -4.45)
	YLDs	86989.50	(66812.41, 110519.75)	127762.69	(100064.06, 157143.12)	46.87	368.76	(289.54, 456.23)	268.41	(209.66, 333.90)	-27.21	-1.02(-1.05,-0.99)
COPD	Incidence	52531.61	(48157.27, 56494.86)	99163.64	(48157.27, 56494.89)	88.77	277.70	(257.78, 294.97)	188.34	(168.48, 208.68)	-32.18	-1.26 (-1.31, -1.20)
	Prevalence	545171.79	(485973.24, 600483.16)	1121545.47	(977516.81, 1277650.14)	105.72	2716.65	(2425.36, 3003.61)	2154.56	(1877.70, 2438.15)	-20.69	-0.75 (-0.79, -0.71)
	Mortality	25958.60	(20876.79, 31103.35)	21028.07	(16055.51, 26777.83)	-18.99	199.75	(162.86, 1236.14)	47.36	(35.86, 60.01)	-76.29	-4.56 (-5.08, -4.03)
	DALYs	543843.28	(445558.45, 647730.40)	398076.01	(325523.76, 488708.73)	-26.80	3344.80	(2744.73, 3955.15)	819.13	(665.92, 1002.76)	-75.51	-4.46 (-4.78, -4.14)
	YLLs	500594.53	(403726.83, 603363.38)	310946.93	(236893.89, 400203.89)	-37.88	3128.87	(2528.57, 3723.87)	651.98	(496.65, 837.23)	-79.16	-4.95 (-5.39, -4.52)
	YLDs	43248.75	(34806.16, 50983.52)	87129.08	(67887.99, 105662.52)	101.46	215.93	(175.70, 215.93)	167.15	(130.05, 200.96)	-22.59	-0.82 (-0.86, -0.78)
Asthma	Incidence	175861.19	(142109.77, 223095.65 )	140464.97	(114339.36, 181977.11)	-20.13	572.28	(468.00, 716.84)	409.61	(324.95, 546.51)	-28.42	-1.09 (-1.26, -0.92)
	Prevalence	1038631.00	(863871.24, 1252428.95)	895335.45	(748131.75, 1072447.61)	-13.80	3642.84	(3123.26, 4306.59)	2285.38	(1860.85, 2833.37)	-37.26	-1.52 (-1.65, -1.40)
	Mortality	1268.11	(903.90, 1856.23)	1010.65	(736.91, 1342.71)	-20.30	8.40	(5.98, 13.44)	2.19	(1.59, 2.91)	-73.93	-4.33 (-4.85, -3.80)
	DALYs	75902.60	(57765.36, 102307.77)	53604.18	(39477.89, 71844.56)	-29.38	313.24	(241.89, 424.64)	128.03	(91.62, 177.74)	-59.13	- 2.85 (-3.12, -2.57)
	YLLs	34671.92	(25557.85, 49887.89)	18372.93	(13676.62, 24700.22)	-47.01	170.28	(123.84, 259.27)	37.35	(28.06, 49.83)	-78.07	-4.86 (-5.33, -4.39)
	YLDs	41230.69	(26220.41, 62348.07)	35231.25	(22285.70, 52556.23)	-14.55	142.96	(91.95, 211.69)	90.68	(56.93, 137.21)	-36.57	-1.51 (-1.62, -1.41)
Pneumoconiosis	Incidence	688.21	(552.66, 794.59)	1024.21	(881.55, 1209.92)	48.82	3.10	(2.60, 3.67)	1.81	(1.57, 2.10)	-41.61	-1.73 (-1.78, -1.68)
	Prevalence	4091.79	(3313.64, 4959.08)	8468.66	(6663.16, 10471.03)	106.97	19.26	(15.70, 23.31)	15.17	(12.01, 18.73)	-21.24	-0.78 (-0.83, -0.73)
	Mortality	234.63	(169.30, 317.88)	292.37	(199.29, 414.43)	24.61	1.22	(0.89, 1.62)	0.56	(0.39, 0.78)	-54.10	-2.54 (-3.05, -2.03)
	DALYs	7502.73	(5538.05, 1007639)	8597.04	(6105.72, 11949.51)	14.59	34.42	(25.69, 45.75)	15.42	(11.05, 21.11)	-55.20	-2.59 (-3.08, -2.09)
	YLLs	6902.03	(4920.16, 9555.76)	7361.87	(4872.75, 10647.08)	6.66	31.62	(22.72, 43.13)	13.21	(8.90, 18.95)	-58.22	-2.85 (-3.19, -2.50)
	YLDs	600.70	(388.57, 857.34)	1820.54	(793.93, 1820.54)	203.07	2.80	(1.82, 4.06)	2.21	(1.43, 3.30)	-21.07	-0.77 (-0.88, -0.65)
ILD PS	Incidence	265.15	(209.03, 323.95)	788.42	(658.91, 928.37)	197.35	1.17	(0.94, 1.41)	1.39	(1.18, 1.62)	18.80	0.56 (0.49, 0.62)
	Prevalence	3314.55	(2525.23, 4162.85)	9357.93	(7563.39, 11384.21)	182.33	15.45	(12.01, 19.08)	16.79	(13.58, 20.29)	8.67	0.27 (0.23, 0.31)
	Mortality	47.10	(26.10, 92.20)	125.56	(76.46, 233.61)	166.58	0.27	(0.15, 0.54)	0.25	(0.16, 0.46)	-7.06	-0.35 (-0.57, -0.13)
	DALYs	1673.83	(1035.32, 2885.41)	3610.64	(2429.14, 6006.88)	115.71	7.78	(4.89, 13.75)	6.74	(4.56, 11.26)	-13.37	-0.50 (-0.73, -0.26)
	YLLs	1319.81	(719.07, 2567.70)	2633.10	(1584.24, 4996.18)	99.51	6.14	(3.41, 12.05)	5.00	(3.02, 9.52)	-18.57	-0.71 (-1.01, -0.41)
	YLDs	354.01	(213.09, 507.97)	977.54	(566.02, 1425.43)	176.13	1.64	(1.00, 2.36)	1.74	(1.02, 2.52)	6.10	0.18 (0.12, 0.24)
other CRD	Mortality	55.74	(31.64, 101.53)	75.63	(54.48, 109.69)	35.68	0.22	(0.13, 0.45)	0.17	(0.12, 0.23)	-22.73	-0.83 (-1.30, -0.35)
	DALYs	5449.23	(3670.16, 8274.31)	5377.40	(4413.17, 6602.26)	-1.32	18.53	(12.51, 28.20)	11.65	(9.60, 14.37)	-37.13	-1.47 (-1.58, -1.36)
	YLLs	3893.89	(2249.52, 6522.96)	2187.76	(1578.70, 3160.08)	-43.82	13.09	(7.53, 22.27)	5.03	(3.71, 6.99)	-61.57	-3.03 (-3.25, -2.81)
	YLDs	1555.34	(1239.05, 1882.84)	3189.64	(2484.86, 3877.20)	105.08	5.44	(4.39, 6.50)	6.62	(5.28, 8.17)	21.69	0.69 (0.51, 0.87)

Notes: CRD: chronic respiratory disease; DALY: disability-adjusted life years; YLD: years of life lost; YLL: years lived with disability;

ASR: age standardized rate; AAPC: average annual percentage change; CI: confidence interval;

COPD: chronic obstructive pulmonary disease; ILDPS:interstitial lung disease and pulmonary sarcoidosis.

### Changes in the CRD burden in different areas

The incidence and prevalence of CRDs showed upward trends worldwide, in China, and in Fujian. The global increase in the incidence and prevalence of CRDs was 10.84% and 22.83%, respectively; the increase in China was 5.36% and 34.29%, respectively; and the increase in Fujian was 5.24% and 28.03%, respectively. The number of CRD-related deaths increased by 47.49% and 3.10% globally and in China, respectively, while it declined by 16.56% in Fujian province. The DALYs and YLLs increased worldwide. In contrast, DALYs and YLLs showed a downward trend in China and Fujian. Compared to 1990, the global YLDs increased by 41.38% in 2021, with YLDs rising by 56.27% and 47.15% in China and Fujian, respectively.

All CRD ASRs showed decreasing trends. The global AAPC of ASIR was −0.88 (95% *CI:* −0.94 to −0.82), with the AAPC of ASIR being −1.02 (95% *CI:* −1.15 to −0.89) and −1.14 (95% *CI:* −1.25 to −1.04) in China and Fujian, respectively. The rate of decline of the incidence of CRDs was faster in Fujian than globally and in China. The ASPR, ASMR, and ASDR for CRDs also demonstrated the same downward trends. In addition, the decline in the AAPC of ASPR, ASMR, and ASDR for CRDs in Fujian was faster than those in the world and China during the same period (Table 4).

**Table 4:** The change of disease burden of CRD in different region, 1990-2021.

Region	Index	Numbers(10^6)				Number Change(%)	ASR(/10^6)				ASR change	AAPC% (95%CI)
		1990		2021			1990		2021			
Global	Incidence	49.81	(42.80, 60.32)	55.21	(48.68, 64.56)	10.84	944.22	(823.94, 1120.03)	719.35	(627.51, 854.14)	-23.82	-0.88 (-0.94,-0.82)
	Prevalence	381.23	(341.23, 513.11)	468.27	(428.93, 513.11)	22.83	7936.44	(7224.91, 8809.07)	5785.36	(5269.67, 6371.88)	-27.10	-1.02(-1.07,-0.97)
	Mortality	2.99	(2.70, 3.20)	4.41	(4.01, 4.87)	47.49	84.55	(76.19, 90.46)	53.56	(48.46, 59.09)	-36.65	-1.47(-1.56,-1.37)
	DALYs	84.89	(76.92, 92.25)	108.50	(76.92, 92.25)	27.81	2075.20	(1893.96, 2237.62)	1294.60	(1196.60, 1412.08)	-37.62	-1.51(-1.58,-1.44)
	YLLs	66.33	(60.08, 70.99)	82.27	(75.46, 90.10)	24.03	1678.78	(1517.02, 1793.11)	975.01	(892.86, 1069.63)	-41.92	-1.73(-1.81,-1.66)
	YLDs	18.56	(13.84, 24.52)	26.24	(20.95, 32.46)	41.38	396.42	(302.19, 508.50)	319.00	(254.46, 401.07)	-19.53	-0.69 (-0.73,-0.65)
China	Incidence	8.02	(6.90, 9.71)	8.45	(7.53, 9.71)	5.36	800.16	(698.65, 950.80)	583.53	(497.86, 714.12)	-27.07	-1.02(-1.15, -0.89)
	Prevalence	56.05	(49.56, 64.15)	75.27	(68.25, 83.78)	34.29	5755.22	(5183.73, 6482.24)	4434.91	(3933.12, 5031,90)	-22.94	-0.84(-0.91, -0.77)
	Mortality	1.29	(1.11, 1.44)	1.33	(1.08, 1.59)	3.10	239.34	(205.82, 239.34)	75.81	(61.99, 89.88)	-68.33	-3.68(-4.02, -3.34)
	DALYs	29.83	(21.88, 30.47)	25.83	(21.88, 30.47)	-13.41	4166.47	(3642.02, 4623.95)	1371.01	(1168.85, 1598.92)	-67.09	-3.56(-3.84, -3.28)
	YLLs	25.88	(22.32, 29.06)	20.72	(16.85, 25.07)	-19.94	3816.92	(3272.56, 4251.87)	1087.99	(888.11, 1307.41)	-71.50	-4.01(-4.32,-3.71)
	YLDs	3.27	(2.54, 4.05)	5.11	(4.06, 6.15)	56.27	349.55	(276.96, 429.49)	283.01	(225.28, 344.74)	-19.04	-0.68 (-0.72, -0.64)
Fujian	Incidence	0.229	(0.195, 0.277)	0.241	(0.211, 0.285)	5.24	854.25	(751.18, 1000.72)	601.15	(511.05, 737.42)	-29.63	-1.14 (-1.25,-1.04)
	Prevalence	1.559	(1.384, 1.784)	1.996	(1.800, 2.241)	28.03	6208.44	(5651.06, 6909.10)	4391.81	(3875.90, 5014.08)	-29.26	-1.12(-1.19,-1.05)
	Mortality	0.028	(0.022, 0.033)	0.023	(0.017, 0.0288)	-16.56	209.87	(170.47, 248.84)	50.53	(38.36, 64.36)	-75.92	-4.51 (-5.04,-3.98)
	DALYs	0.634	(0.525, 0.759)	0.469	(0.383, 0.057)	-26.07	3718.76	(3079.45, 4389.72)	980.97	(804.50, 1195.49)	-73.62	-4.23(-4.52,-3.93)
	YLLs	0.547	(0.442, 0.660)	0.342	(0.261, 0.440)	-37.48	3350.00	(2707.64, 4000. 28)	712.56	(544.04, 914.98)	-78.73	-4.89(-5.33, -4.45)
	YLDs	0.087	(0.067, 0.111)	0.128	(0.100, 0.157)	47.15	368.76	(289.54, 456.23)	268.41	(209.66, 333.90)	-27.21	-1.02(-1.05,-0.99)

Notes: CRD: chronic respiratory disease; DALY: disability-adjusted life years; YLD: years of life lost; YLL: years lived with disability;

ASR: age standardized rate; AAPC: average annual percentage change; CI: confidence interval.

### Attribution of major risk factors of CRDs

Among the risk factors identified by GBD 2021, smoking was the most important contributor to DALYs for CRDs, accounting for 352.05 (95% *CI:* 242.12–473.24) per 100,000 people. The AAPC of ASDR of smoking between 1990 and 2021 was −4.30% (95% *CI:* −4.88 to −3.71), accounting for 35.82% (95% *CI:* 27.49–43.01) of the attributable risk for CRD DALYs in Fujian province. Ambient PM pollution ranked as the second most important risk factor, accounting for 17.51% (95% *CI:* 13.88–20.89) of CRD DALYs, and its AAPC of ASDR of ambient PM pollution was −2.44% (95% *CI:* −2.78 to −2.11). Occupational exposure ranked as the third most important factor, accounting for 14.57% (95% *CI:* 11.74–17.26) of CRD DALYs, and its AAPC of ASDR was −4.47% (95% *CI:* −4.88 to −4.06).

**Fig 1:**
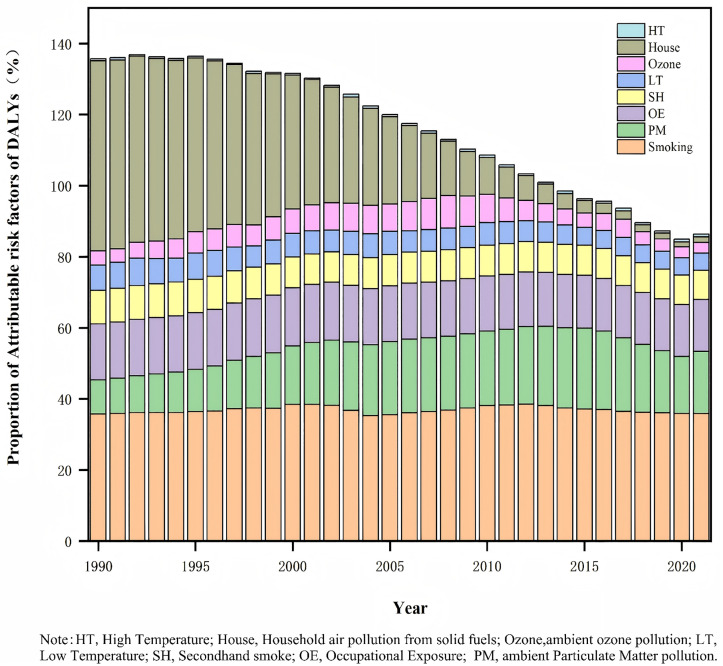
The proportion of attributable risk factors of DALYs of CRD in Fujian, 1990-2021. Legend: The bar charts showed the proportion of eight attributable risk factors of DALYs of CRD in Fujian. The height of each bar represented the percentage of DALYs for the risk factors, *95% CIs* were not shown.

During these 32 years, the contribution of household air pollution from solid fuels to CRD DALYs decreased the most. In 1990, household solid fuels contributed 1994.40 (95% *CI:* 1474.58–2502.64) per 100,000 people CRD DALYs, accounting for 53.60% (95% *CI:* 43.67–61.73) of CRD DALYs in 1990. However, in 2021, it accounted for only 15.52 (95% *CI:* 0.14–124.47) per 100,000 people and only 1.59% of CRD DALYs (95% *CI:* 0.01–13.05). The AAPC of ASDR between 1990 and 2021 was −14.68% (95% *CI:* −14.96 to −14.40). The proportion of DALYs attributed to various factors, including smoking, occupational exposure, secondhand smoke, low temperatures, ozone pollution, and high temperatures, remained stable over 32 years (Table 5 and Fig 1).

**Table 5:** The change of attribute risk factors of DALYs in CRD in Fujian, 1990-2021.

Population	Risk factors	Proportion (%)				ASDR (1/10^6)				
		1990		2021		1990		2021		AAPC (95%*CI,%*)
Total	Smoking	35.68	(28.51, 42.93)	35.82	(27.49, 43.01)	1326.06	(1013.12, 1687.99)	352.05	(242.12, 473.24)	-4.30 (-4.88, -3.71)
	Ambiet PM pollution	9.64	(4.27, 17.90)	17.51	(13.88, 20.89)	357.54	(159.44, 695.51)	171.94	(122.72, 228.22)	-2.44 (-2.78, -2.11)
	Occupational Exposure	15.76	(13.15, 18.36)	14.57	(11.74, 17.26)	585.81	(453.32, 720.24)	143.19	(102.79, 188.12)	-4.47 (-4.88, -4.06)
	Secondhand Smoke	9.44	(3.82, 15.16)	8.28	(3.39, 13.04)	350.63	(135.64, 582.07)	81.34	(33.71, 136.21)	-4.63 (-5.00, -4.25)
	Low Temperature	7.09	(5.42, 8.68)	4.83	(3.75, 5.85)	263.90	(194.84, 345.21)	47.49	(34.18, 64.11)	-5.60 (-6.83, -4.35)
	Ambiet Ozone pollution	3.95	(0.60, 8.14)	2.95	(0.05, 6.12)	147.14	(23.18, 291.33)	28.92	(4.49, 60.92)	-5.12 (-6.89, -3.32)
	Household pollution	53.60	(43.67, 61.73)	1.59	(0.01, 13.05)	1994.40	(1474.58, 2502.64)	15.52	(0.14, 124.47)	-14.68 (-14.96, -14.40)
	High Temperature	0.05	(-0.83, 2.80)	0.81	(-0.10, 3.57)	19.21	(-30.97, 107.33)	7.91	(-9.36, 35.42)	-3.20 (-8.04, 1.89)
Males	Smoking	6.18	(0.52, 0.70)	61.81	(52.19, 70.10)	3068.89	(2372.19, 3963.04)	786.10	(532.06, 1052.32)	-4.39 (-4.90, -3.89)
	Ambiet PM pollution	11.23	(4.93, 20.86)	17.76	(14.26, 21.28)	556.21	(245.66, 1073.27)	241.94	(165.82, 324.83)	-2.75 (-3.49, -2.01)
	Occupational Exposure	18.83	(14.79, 23.16)	16.73	(12.35, 21.14)	935.99	(658.21, 1225.26)	228.03	(148.69, 317.97)	-4.54 (-5.04, -4.04)
	Secondhand Smoke	6.45	(2.47, 10.72)	6.26	(2.42, 10.45)	319.29	(127.32, 535.75)	85.34	(29.68, 149.78)	-4.27 (-4.68, -3.85)
	Low Temperature	7.29	(5.59, 8.94)	5.45	(4.25, 6.47)	362.62	(252.86, 480.47)	74.35	(50.94, 103.37)	-5.15 (-7.03, -3.24)
	Ambiet Ozone pollution	4.06	(0.63, 8.33)	3.33	(0.51, 6.84)	201.71	(32.79, 411.94)	45.36	(6.76, 99.91)	-4.71 (-7.05, -2.31)
	Household pollution	51.43	(41.07, 60.76)	1.36	(0.01, 11.00)	2556.38	(1809.36, 3370.42)	18.60	(0.16, 163.20)	-15.02 (-15.67, -14.36)
	High Temperature	0.53	(-0.87, 2.91)	0.91	(-1.06, 4.04)	26.29	(-45.76, 147.42)	12.33	(-14.32, 54.72)	-2.97 (-5.24, -0.64)
Females	Ambiet PM pollution	7.97	(3.55, 14.98)	17.36	(13.70, 20.88)	231.36	(98.89, 451.47)	123.34	(85.74, 177.29)	-2.13 (-2.95, -1.31)
	Secondhand Smoke	13.11	(5.49, 17.93)	11.56	(4,86, 17.93)	381.48	(149.31, 640.24)	82.08	(32.78, 137.58)	-4.86 (-5.33, -4.39)
	Occupational Exposure	12.27	(9.12, 15.70)	11.35	(8.35, 14.91)	356.60	(236.05, 500.06)	80.63	(54.47, 121.75)	-4.70 (-5.27, -4.12)
	Smoking	6.74	(3.78, 10.99)	4.38	(2.36, 7.68)	196.16	(100.87, 349.92)	31.12	(15.85, 59.69)	-5.70 (-6.47, -5.10)
	Low Temperature	6.88	(5.21, 8.43)	3.98	(2.88, 5.04)	200.29	(126.67, 279.92)	28.43	(18.26, 43.19)	-6.22 (-7.81, -4.59)
	Ambiet Ozone pollution	3.83	(0.06, 7.96)	2.42	(0.04, 5.15)	112.00	(17.47, 235.02)	17.27	(2.50, 38.52)	-5.60 (-8.29, -2.84)
	Household pollution	56.21	(47.37, 63.86)	1.95	(0.02, 16.23)	1634.86	(1156.78, 2160.05)	13.55	(0.13, 105.85)	-14.51 (-15.36, -13.65)
	High Temperature	0.50	(-0.80, 2.68)	0.07	(-0.80, 3.03)	14.64	(-22.48, 85.04)	4.78	(-5.81, 21.73)	-4.00 (-8.87, 1.14)

Notes: CRD: chronic respiratory disease; DALY: disability-adjusted life years; YLD: years of life lost; YLL: years lived with disability;

ASDR: age standardized DALY rate; AAPC: average annual percentage change; CI: confidence interval.

95% CI: 4.86–17.93), and occupational exposure ranked third (11.35%, 95% CI: 8.35–14.91), all showing downward trends (Table 5).

Stratifying by sex, the ranking of risk factors among men was the same as that of the entire population. In contrast, the ranking of risk factors among women was different, with ambient PM pollution ranked first (17.36%, 95% *CI*: 13.70–20.88), secondhand smoke ranked second (11.56%,

## Discussion

In this study, we utilized the data from the GBD 2021 to examine the burden of CRDs in Fujian province. The incidence and prevalence of CRDs in Fujian increased over the past 32 years. However, the ASIR, ASPR, ASMR, and ASDR for CRDs all showed significant downward trends. Similar to the changing trends of CRDs studied at home and abroad, the burden of CRDs in Shanghai [9] and Jiangsu province [10] also showed downward trends. Previous studies have primarily reported the burden of CRDs at regional and national levels [11], while few studies have reported provincially representative data. Moreover, all indicators of disease burden were in Fujian than in the world and China.

As we know, after age-standardized by the global standard population, the age-structure differences have been removed. Thus, the age-standardized rate can be used to determine whether the true risk of CRD has changed. The main reasons for the significant decline in the overall CRD burden in recent years include improvement in economic and living standards, advancement in the management of medical conditions, and the availability of sufficient health resources, as well as increased health awareness among the public. The above have played positive roles in the prevention, diagnosis, and treatment of CRDs [10]. Due to the aging population, the incidence and prevalence of CRDs will continuously increase. Although the ASMR for CRDs is declining, CRDs are still ranked as the fourth leading cause of death in Fujian [12]. They remain predominant chronic diseases with a relatively heavy disease burden in Fujian province and require the attention of the government and society.

Effective CRD prevention and control measures should be applied to different age groups and sexes. Males bear a heavier disease burden than females. In 2021, the ASDR and ASYLLR for CRDs among male residents in Fujian province were 1360.18/100,000 and 1117.26/100,000, respectively, which were higher than the 708.56/100,000 and 420.59/100,000 among females. These findings are consistent with those of foreign and Chinese studies (9–11). The prevalence and burden of CRDs are greater among men, which may be related to the highly prevalent smoking behavior and occupational exposure to chemical dust and harmful gases among men [13]. Tobacco exposure in Fujian was higher among men. The current smoking rate among males is 56.2%, which is significantly higher than that of females (0.7%) [13]. Furthermore, the occupational dust and harmful gases exposure rate among males (43.4%) was also higher than that in females (29.4%) [13]. However, the ASYLDR among females was 287.97 per 100,000, which was higher than that among males (242.92 per 100,000). The finding indicates that women experienced more severe disability, which may be related to their longer life expectancy, higher frequency of exposure to cooking exhaust gas, and secondhand smoke [13]. Particular attention should be paid to the disease burden due to disability.

The burden of CRDs increases significantly with age. The CRD-related mortality rate among those under 49 years tended to be low and stable. Conversely, the mortality rate among people aged over 50 years increased significantly. This upward trend was remarkable and reached a peak in the group above 70 years old. This trend may be related to increasing age and the accumulation of multiple risk factors, resulting in organ decline and functional degradation [14]. CRDs have led to a decrease in the quality of life, disability, and increasing medical burden. Long-term CRDs can cause deterioration in lung function, loss of working ability, and the need for long-term care by family members, affecting work, creating other practical problems, and adding a heavy economic burden to families and society [4]. Residents in Fujian have relatively limited knowledge and awareness about CRDs [13]. High-risk groups and patients with CRDs have no obvious symptoms at the early stage of the disease. Even when they have symptoms, they do not receive sufficient attention and may not seek professional prevention and treatment early. Therefore, they often miss the best intervention period [15]. These results suggest that middle-aged and older people are the key population to be screened for CRDs, and they should be considered the high-risk population for CRDs.

COPD and asthma are the two main types of CRD, affecting the highest proportion of patients and making the greatest contribution to the burden of CRDs. Most patients with asthma are diagnosed at a young age. With the advancement in medicine, environment improvement and economic developed during these 32 years, the prevalence and ASRs of asthma are decreasing. This may be attributed to the improvement in asthma management through the introduction of inhaled corticosteroids (ICS) or a combination therapy of ICS with long-acting beta-agonists [16]. Moreover, the change in public policy and social economics may have played important roles in improving air quality, reducing tobacco usage, and creating awareness of early screening for asthma, which have influenced disease rates [17,18].

The number of patients with COPD and ILDPS significantly increases aging, and the number of patients with pneumoconiosis increased with industrialization level with a lack of necessary protective methods and healthy practices [13]. Most of the ASRs exhibited decreasing trends, with only the ASIR and ASPR for ILDPS showing an increase. This may be due to population aging, workplace exposure to dust, gases, heavy metals, and other pollutants, and the use of more advanced medical investigations, such as high-resolution CT, for lung disease evaluation and pulmonary examination [19], which might impact the progress of these chronic respiratory diseases. Moreover, these diseases are not curable, numberous treatment and medicine could only help to control clinical symptoms and improve life quality of CRD patients [11], leading to the rising trends in incidence and prevalence rates.

The early clinical symptoms and signs of CRDs are not obvious and can be easily ignored; therefore, patients and populations at high risk of CRDs may not identify these diseases early. Additionally, once these diseases persist and gradually deteriorate, they may become irreversible, imposing significant physical and economic burdens on patients, their families, and society [4]. Missed diagnosis and misdiagnosis of CRDs persists in China and Fujian province, which might lead to an underestimation of the actual number of cases and deaths due to CRDs [20]. Pulmonary function tests should be popularized among older persons as early as possible. Corresponding measures, such as health education of high-risk groups, timely diagnosis and treatment of patients, encouraging patients to use inhalation drugs regularly, undergoing respiratory function recovery training, and improving nutritional support for patients, should be taken when high-risk groups are identified to reduce the burden of CRDs [10].

The attributable risk factors were ranked as follows: smoking, ambient PM pollution, and occupational exposure. These are the top three risk factors for CRDs in Fujian. The GBD attributed to ambient PM pollution is the highest, accounting for approximately 8.0% (95% *CI*: 6.7–9.4) of the total DALYs. Smoking accounts for 5.7% (95% *CI*: 4.7–6.8) of DALYs globally [21]. This global ranking differs slightly from the ranking of risk factors of CRDs in Fujian province.

More than 1.14 billion people are current smokers, and the smoking prevalence exceeds 20% globally [22]. The smoking rate among people aged 40 and above was 44.8% (males 80.0%, females 2.5%) in Fujian province in 2015, the smoking rate was 42.4% (males 78.5%, females 0.9%) in Fujian province in 2020 [13]. The smoking rate decreased, however, the smoking behaviour remained highly popular [15]. Smoking has been repeatedly confirmed to be closely related to the occurrence and mortality due to various chronic diseases, such as ischemic heart diseases, lung cancer, and respiratory diseases [22]. Tobacco smoke not only influences the pathogenesis of COPD, pneumoconiosis, and ILDPS but is also the main risk factor that impacts the progress of asthma [16]. The exposure of adults to secondhand smoke in Fujian is also not encouraging. Surveys indicate that 77.2% of residents reported they were exposed to secondhand smoke daily [23]. It indicated that tobacco use and smoke exposure were still widespread in the daily lives of Fujian residents [23]. To reduce the demand for tobacco products, coercive and restrictive measures were adopted, including raising tobacco tax rates [22], clarifying health warning requirements through legislation, creating more smoke-free working environments and public places, and strictly prohibiting tobacco advertising.

With rapid economic development and industrialization, environmental PM pollution and occupational exposure have become public problems. Waste gas from the combustion of various fuels in industries, exhaust gas from vehicles, and chemical dust and gases in working places contain toxic dust and other harmful substances. Air pollution, primarily consisting of PM_10_ and PM_2.5_, stimulates the human respiratory tract and lungs, increasing the risk of CRDs. Moreover, long-term and repeated exposure to these pollutants increases the risk of adverse health outcomes [24]. These air pollutants led to increasing trends in the number of new cases, prevalence, deaths, and DALYs due to pneumoconiosis and ILDPS in the GBD 2021. Occupational exposure rate among middle-aged and elderly population was 50.0% in Fujian residents in 2015, 56.1% in males and 39.7% in females. This rate decreased to 36.8% in Fujian population in 2020, 43.4% in males and 29.4% in females, with decreasing trends in both gender [13]. Environmental and health departments need to strengthen efforts to improve air quality and working environment. It is also necessary to strengthen early warning and forecasting of extreme weather and environmental pollutants to improve the living environment of Fujian residents.

The most remarkable decline among all the risk factors was in household air pollution from solid fuels. Many farmers easily collected wood and crops as free fuels for cooking and heating from the natural environment in 1990. With improvements in the economy and health awareness, an increasing number of people have accepted the concepts of environmental protection and a healthy lifestyle. Consequently, the consumption of biomass fuels in rural areas in China has decreased by more than 50%, and individuals have made the right choice of transitioning from using polluted energy to using environmentally friendly energy during the last three decades [25,26]. With the effectiveness of environmental protection policies and economic development, Fujian residents shifed biomass fuel consumption into clean energy usage, reducing the health and economic impacts of pollution. The proportion of household polluting fuel for cooking and heating was 37.2% and 3.5% in 2015, the data declined to 25.2% and 0.8% in 2020 in Fujian province in a decling trend [13].

Recently, the Chinese government realized the serious impact of CRD and has focused on several regular screening and intervention projects for high-risk populations and patients with CRDs in grassroots medical and health institutions [7]. Comprehensive and multi-dimensional health education on disease prevention and behavioral intervention was also improved for the public. The government has taken multiple measures to enhance public awareness, including regular follow-up physical examinations, the introduction of early symptoms and signs of CRDs, recommendations for pulmonary function tests and healthy lifestyles for high-risk populations, the use of new media publicity channels, and increasing the frequency and duration of information promotion.

This study was the first to evaluate the burden of CRDs in Fujian between 1990 and 2021 at the provincial level, and its results reflected the impact of CRDs among Fujian residents. The results of this study provide scientific support for informed prevention policy and effective government decision-making. Our study has several limitations. First, we only estimated the burden of CRDs at the provincial level. We did not divide the province into smaller sections at the city or county level. More detailed studies on the burden of CRDs by district and city or by urban and rural areas should be performed in the future. Second, because the GBD 2021 data are derived from multiple health information systems in China, the accuracy and reliability of the data rely on the coverage of monitoring sites and the quality of the original data in various programs in China. Third, the GBD method employed statistical models to evaluate and predict data under ideal conditions, which introduces some uncertainty and bias; therefore, the results should be interpreted carefully in the real world. In future studies, more efforts should be made to obtain more accurate and accessible epidemiological data for GBD research.

## Conclusion

Our study revealed that the overall CRD burden in Fujian province decreased at the provincial and regional level between 1990 and 2021. The ASIR, ASPR, ASMR, and ASDR for CRDs among Fujian residents generally showed downward trends. However, the incidence and prevalence of CRDs increased. Therefore, CRDs remain an important public health problem in Fujian. Health education programs to prevent and control CRDs should target middle-aged and older men. Controlling smoking, protecting the environment from air pollution, and preventing occupational exposure should be prioritized in future public health policies in Fujian.

## Abbreviations

CRD: chronic respiratory disease
COPD: chronic obstructive pulmonary disease
ILDPS: interstitial lung disease and pulmonary sarcoidosis
GBD: global disease burden
DALY: disability-adjusted life years
YLL: years of life lost
YLD: years lived with disability
ASIR: age standardized incidence rate
ASPR: age standardized prevalence rate
ASMR: age standardized mortality rate
ASDR: age standardized DALY rate
APC: annual percentage change
AAPC: average annual percentage change
CI: confidence interval
UI: uncertainty interval.
